# Predictors of Revascularization in Patients with Unstable Angina

**DOI:** 10.3390/jcm13041096

**Published:** 2024-02-15

**Authors:** Jan Budzianowski, Wojciech Faron, Janusz Rzeźniczak, Marek Słomczyński, Dariusz Hiczkiewicz, Jacek Olejniczak, Jarosław Hiczkiewicz, Paweł Burchardt

**Affiliations:** 1 “Club 30”, Polish Cardiac Society, 93-338 Łódź, Poland; pburchardt@ump.edu.pl; 2 Department of Interventional Cardiology and Cardiac Surgery, Collegium Medicum, University of Zielona Góra, 65-046 Zielona Góra, Poland; dhiczkiewicz@uz.zgora.pl (D.H.); jhiczkiewicz@uz.zgora.pl (J.H.); 3 Department of Cardiology, Nowa Sól Multidisciplinary Hospital, 67-100 Nowa Sól, Poland; wfaron@poczta.onet.pl; 4 Department of Cardiology, J. Strus Hospital, 61-285 Poznań, Poland; syngo@wp.pl (J.R.); mslomczynski@interia.pl (M.S.); 5 J. Strus Hospital, 61-285 Poznań, Poland; jacek.olejniczak16@gmail.com; 6 Department of Hypertension, Angiology, and Internal Medicine, Poznan University of Medical Sciences, 61-848 Poznań, Poland

**Keywords:** acute coronary syndrome, percutaneous coronary intervention, unstable angina, coronary artery bypass graft

## Abstract

Background: The factors that determine the necessity of coronary artery revascularization in patients with unstable angina (UA) have been supported by limited data. Therefore, this study aimed to identify the predictors of revascularization in patients with UA. Methods: The study included the recorded data of 3668 patients with UA who underwent cardiac catheterization (age 66 ± 9.2, men 70%); 2615 of them (71%) underwent revascularization (percutaneous transluminal coronary angioplasty (PTCA), coronary artery bypass graft (CABG), or hybrid revascularization. The remaining 1053 patients (29%) had no significant coronary stenosis and were regarded as controls. Multivariable logistic regression analysis was performed to separate the predictors of revascularization. Results: It was found that severe angina (OR 2.7, 95%CI 1.9–3.7), male gender (OR 1.4, 95%CI 1.1–1.7), and hyperlipidemia were the predictors of revascularization. It was also noted that intraventricular conduction disorders including left and right bundle branch blocks and a history of previous revascularization and myocardial infarction were associated with lower odds of revascularization. Conclusion: Overall, however, the predictive value of the studied factors proved to be poor and may still point to the multifactorial nature of significant coronary artery stenosis and the need for revascularization in patients with UA.

## 1. Introduction

It is estimated that 10% of patients with acute coronary syndromes (ACS) suffer from UA, known as a clinical condition characterized by myocardial ischemia at rest or during minimal exertion without acute myocardial necrosis/injury. It is also characterized by specific clinical findings, including prolonged (>20 min) angina at rest; angina that is escalating in frequency or duration, or lowering in threshold; the sudden onset of severe angina; or angina that occurs after a recent episode of MI [1]. UA and myocardial infarction belong to the ACS spectrum, given the close pathophysiological link between them (plaque erosion plays a significant role in both) [2].

The first record of UA appeared in 1937 when Sampson, Eliaser, and Feil described several patients with prolonged, severe anginal pain that preceded the occurrence of myocardial infarction [3,4]. The term unstable angina, coined by Fowler and Conti in 1971, marked a significant event in the history of ACS [5,6]. The term was described as a continuum between stable angina and myocardial infarction.

Noteworthily, although the clinical characteristics of UA have persisted unchanged over time, the biomarkers of myocardial necrosis have undergone fundamental evolution. This clarifies the enduring challenge in the clinical diagnosis and management of UA that patients have been facing over the years. For example, in most UA patients, their troponin levels will be below the 99th percentile. However, there are still recorded cases of patients diagnosed with UA who have elevated troponin levels and a pattern that does not change [7].

The key element for differentiating between UA and Non-ST-segment elevation myocardial infarction (NSTEMI) is the assessment of the levels of cardiac injury biomarkers. In the 1980s and 1990s, the MB fraction of creatine kinase (CK-MB) and first-generation cardiac troponin (cTn) assays were routinely applied. However, both lacked optimal sensitivity and specificity for detecting myocardial necrosis. The very high incidence of UA in the 1990s therefore appears to have been an overestimate [8].

The introduction of an assay for cardiac-specific troponin I and T provided a more sensitive and specific marker than CK-MB for detecting myocardial necrosis more frequently and more accurately [9,10]. This assay became a breakthrough in the classification of patients with ACS. Additionally, the introduction of improved high-sensitivity (hs) cTn assays led to an increase in the detection of NSTEMI and a reciprocal decrease in the diagnosis of UA. This has also influenced our comprehension of previously established risk stratification strategies [7,11,12]. The more accurate high-sensitive troponin assays revealed a decrease in the prevalence of UA from 35–61% to 7–9% [13,14,15]. It was then shown that an increase in hsTnT had prognostic value. A hsTnT value above the upper reference level (URL) is associated with a twofold increase in cardiovascular death or myocardial infarction (MI) within 1 year [16]. Conversely, low levels of troponins on admission allow for the early and safe discharge of over two-thirds of patients with suspected ACS [17].

Despite advancements in the tools assisting in detecting UA, a few issues still need to be addressed to improve UA diagnosis and therapeutic strategy. For example, in many clinical studies, UA and NSTEMI are still commonly classified together as non-ST-segment elevation ACS (NSTE-ACS), given that there are few analyses of patients with UA only [18]. There is also a lot of uncertainty in the management of patients with UA, as evidence for the benefits of an invasive strategy within 72 h is low [19,20]. Moreover, the correct qualification of UA patients for adequate interventional treatments is still problematic, as the amount of PCI in UA is lower than even in stable coronary artery disease (CAD) [21]. Additionally, the diagnosis of UA remains challenging, given the subjective assessment of index symptoms; this may cause a high risk of bias, leading to the frequently overused diagnosis of UA becoming an indication for urgent CA. The question of which UA patients should be qualified for early invasive strategies remains unresolved.

Lastly, as mentioned before, the data indicating the parameters that determine the presence of significant stenosis in UA patients (and, consequently, clarifying their PCI performance) is still limited. Hence, the low percentage of PCI in patients with UA motivated this study. It was the study’s objective to look for the clinical factors contributing to significant coronary artery stenosis.

## 2. Materials and Methods

### 2.1. Study Population

This single-center retrospective study included 3668 consecutive adult patients (≥18 years old) with diagnosed UA hospitalized in the Department of Cardiology in Multidisciplinary Hospital Nowa Sól, Poland between January 2012 and December 2016.

UA was defined as myocardial ischemia at rest or during minimal exertion in the absence of acute cardiomyocyte injury/necrosis, based on the contemporary TnI and high sensitivity Troponin T (hs-TnT) level (below 99th percentile URL) in the cTn-assay used [1,22]. A 0 h/3 h algorithm assay was applied along with clinical and ECG findings to rule out NSTEMI. When classifying angina symptoms, an internal questionnaire was devised in which the patient was asked about clinical symptoms. Anginal pains were classified as: Class I—mild, Class II—moderate, Class III—severe, and Class IV as very severe.

When assessing stenosis, quantitative coronary angiography (QCA) and the procedural expertise of a skilled cardiologist were employed. Notably, a reduction in the diameter of epicardial arteries exceeding 70% (by contemporary standards) was deemed significant, warranting revascularization [1]. In instances where ambiguity persisted, fractional flow reserve (FFR) evaluations were conducted, adhering to the guidelines outlined by the European Society of Cardiology (ESC). It is noteworthy that the utilization of FFR was sporadic, primarily due to the limited availability of the procedure in Poland during the implementation of the project.

The patients were qualified for diagnostic CA based on the algorithm for NSTE-ACS management. CA was performed in all 3668 patients. In the case of 2615 patients, coronary revascularization (PCI, CABG) was carried out due to the presence of significant coronary artery stenoses. In the remaining 1053 individuals, the presence of significant stenoses was not observed, and they were not qualified for revascularization. They were considered the control group. The data were collected from electronic health records.

The management of the study population followed the current recommendations of the European Society of Cardiology. The study was retrospective, conducted in adherence to the principles of the Helsinki Declaration, and did not necessitate a separate approval from the bioethics committee. Exclusion criteria involved: patients with incomplete documentation and laboratory exams; patients with significant coronary artery stenoses (who had not undergone CABG previously) eligible for conservative treatment; patients who were not qualified for either CABG or PCI due to technical issues.

### 2.2. Laboratory Assessments

The blood samples were obtained at baseline. Venous blood was drawn from the basilic vein. In the analysis, the peripheral blood count was marked with CELL-DYN Ruby (Abbott Diagnostics, Santa Clara, CA, USA). Fibrinogen, D-dimer, aPTT and INR were determined using STACompact Max (Diagnostica Stago, Parsippany, NJ, USA). Creatinine, total cholesterol, high-density lipoproteins, and triglycerides were analyzed using a photometric test (Roche Diagnostics GmbH, Mannheim, Germany).

The assessment of cardiac troponin involved the use of two assays: (a) a traditional immunoassay technique carried out from January 2012 to September 2014 (TnI-Ultra from Siemens, Advia Centaur, Deerfield, IL, USA), featuring a limit of detection of 6 pg/mL (0.006 ng/mL), a 99th percentile reference limit of 40 pg/mL (0.04 ng/mL), and a total imprecision of 10% at a concentration of 30 pg/mL (0.03 ng/mL) [23]; (b) the blood samples taken between October 2014 and December 2016 were analyzed by high sensitivity assays for cTnT (Roche Diagnostics, Basel, Switzerland), which have a limit of detection at 5 ng/L and a 99th percentile reference limit of 14 ng/L, with a total imprecision of 10% at a concentration of 13 ng/L.

### 2.3. Statistical Analysis

Continuous variables with a normal distribution were presented as mean and standard deviation. Non-normal variables were reported as the median and interquartile range. Student’s *t*-test was used to test the significance of the assessed parameters between the two groups in the case of variables with a normal distribution. For the variables that were not normally distributed, the Mann–Whitney test was employed to compare the groups. The frequencies of categorical variables were compared using the Chi-square or Fisher’s exact test when appropriate. Aiming to determine the cut-off point of the predictors for significant coronary artery stenosis in patients with UA, a receiver-operating characteristic (ROC) curve was created. The cut-off points of the analyzed predictors, (which differentiated the patients with PCI + CABG from the patients with a hybrid treatment), were estimated based on the Youden index, and their quality was assessed using two indicators: sensitivity and specificity. A logistic regression model was used to assess the need for PCI + CABG or hybrid treatment.

Subsequently, one-dimensional logistic regression models were applied, followed by the multi-dimensional logistic regression model (MLR). The MLR model was constructed using the backward stepwise logistic regression method. The results obtained were presented as an odds ratio (OR) with a 95% confidence interval (CI). Statistical analysis was performed using Statistica 13.3 software (StatSoft, Tulsa, OK, USA). All the tests were analyzed at alpha significance level = 0.05.

## 3. Results

### 3.1. Patient Characteristics

**Figure 1:**
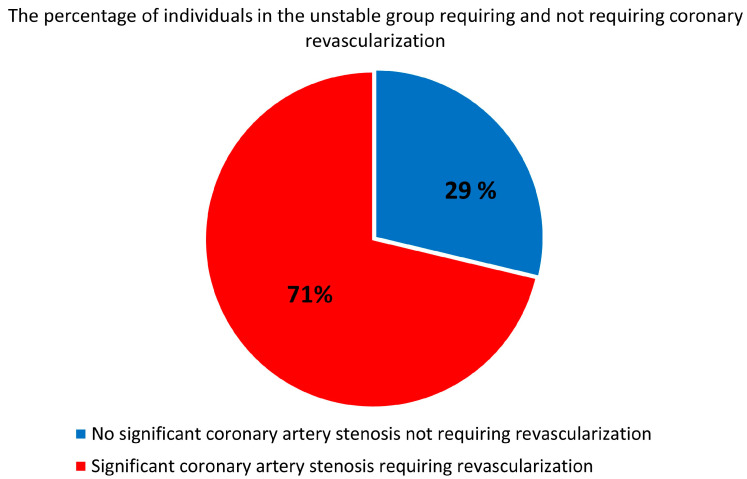
The percentage of UA individuals requiring and not requiring coronary revascularization.

All 3668 patients with diagnosed UA underwent CA; 2615 (71%) patients underwent revascularization due to significant coronary stenosis (Figure 1). The mean age was 66 ± 9.2.

Table 1 presents a summary of the baseline characteristics and differences observed between the coronary artery group requiring revascularization and the control group. The patients who underwent revascularization due to significant coronary stenoses were more likely to have a higher prevalence of cardiovascular risk factors (such as total cholesterol, LDL-cholesterol, and lower HDL levels) compared to patients without obstructive coronary artery disease who did not require revascularization (Table 1). Moreover, symptoms of stenocardial pain of class III were more likely in the revascularization group. However, the percentage of previous PCI, CABG, a history of myocardial infarction, and the presence of LBBB and RBBB were higher in the control group (Table 1).

### 3.2. Predictors of Revascularization in UA—Multivariate Analysis

The multivariate logistic regression model was built using the backward stepwise logistic regression method. Stenocardial pain of class III, male sex, total cholesterol concentration above 155 mg%, LDL concentration above 87 mg%, and platelet count above 210.000/mL were the independent predictors of revascularization in the study group. Additional parameters that characterized the patients in this analysis included: an MCV less than 90.9 fl, prothrombin time (PT) less than or equal to 14.2 s, RDW less than 11.6%, and TSH less than 1.05 IU/mL (Table 2). Moreover, patients who underwent revascularization less frequently had LBBB and RBBB at baseline ECG.

### 3.3. Predictors of Revascularization in Patients with UA—ROC Curves

**Figure 2:**
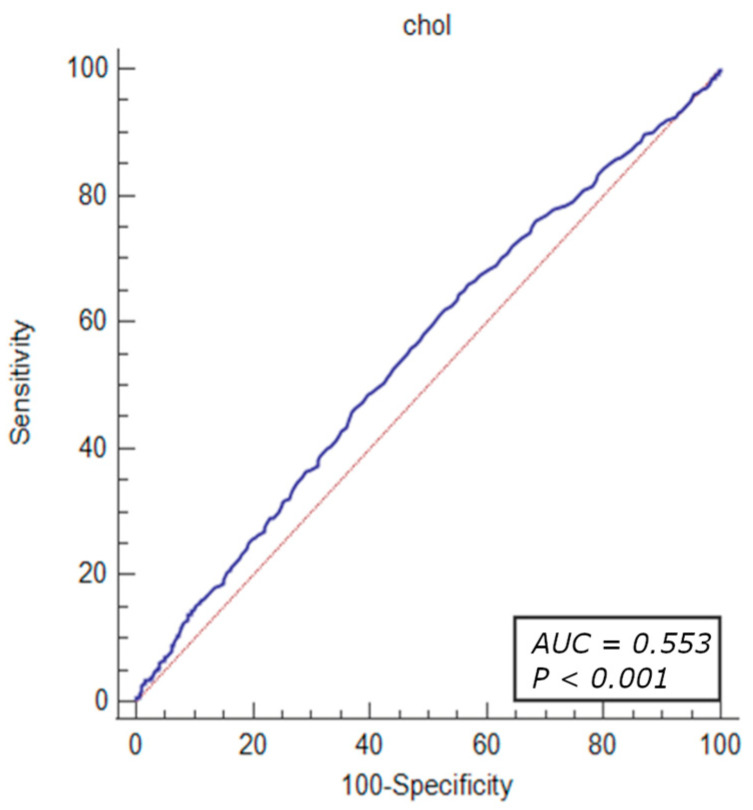
ROC curves of total cholesterol levels for the diagnostic ability of revascularization in UA. AUC for total cholesterol (blue line), reference line (red line).

**Figure 3:**
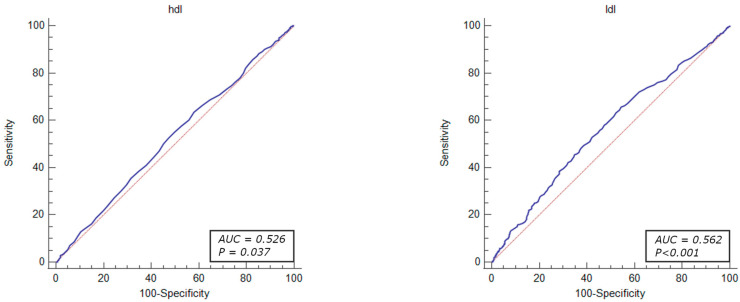
ROC curves of HDL and LDL-cholesterol levels for the diagnostic ability of revascularization in UA. AUC for HDL and LDL cholesterol (blue lines), reference lines (red lines).

**Figure 4:**
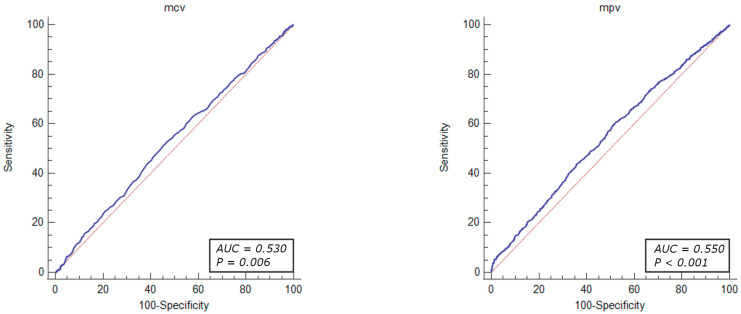
ROC curves of MCV and MPV for the diagnostic abilities of revascularization in UA. AUC for MCV and MPV (blue lines), reference lines (red lines).

**Figure 5:**
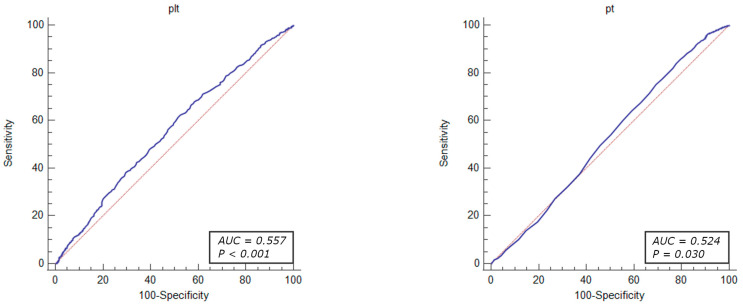
ROC curves of platelets and prothrombin time for the diagnostic abilities of revascularization in UA. AUC for PLT and PT (blue lines), reference lines (red lines).

**Figure 6:**
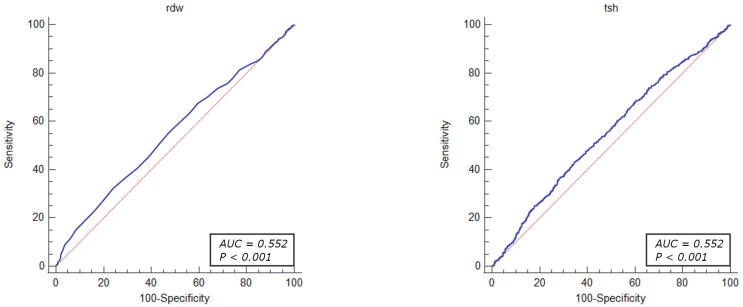
ROC curves of RDW and TSH for the diagnostic abilities of revascularization in UA. AUC for RDW and TSH (blue lines), reference lines (red lines).

The diagnostic value of the factors associated with revascularization due to significant coronary stenosis were evaluated through ROC curve analysis. The results demonstrated that the total cholesterol levels > 155 mg/dL (OR = 1.46, 95% CI: 1.23–1.74, *p* < 0.001), HDL-cholesterol level < 54 mg/dL (OR = 1.25, 95% CI: 1.05–1.49, *p* = 0.010), LDL-cholesterol level > 87 mg/dL (OR = 1.61, 95% CI: 1.35–1.91, *p* < 0.001), MCV < 90.9% (OR = 1.26, 95% CI: 1.08–1.46, *p* = 0.002), MPV < 8.8% (OR = 1.40, 95% CI: 1.21–1.63, *p* < 0.001), PLT levels > 210 × 10^3^ mL (OR = 1.48, 95% CI: 1.27–1.71, *p* < 0.001), prothrombin time < 14.2 s (OR = 1.53, 95% CI: 1.26–1.85, *p* < 0.001) RDW level < 11.2% (OR = 1.48, 95% CI: 1.27–1.79, *p* < 0.001), and TSH level < 1.05 µU/mL (OR = 1.42, 95% CI: 1.20–1.68, *p* < 0.001) were identified as independent predictors of revascularization attributed to significant coronary artery stenosis in patients with UA (Figure 2, Figure 3, Figure 4, Figure 5 and Figure 6).

## 4. Discussion

Most ACS events are caused by the rapture or erosion of an atheromatous plaque that is not angiographically critical. The angiographic extent and severity of coronary artery stenosis are strongly associated with survival [24]. SYNTAX and Gensini scores, established to define the severity of coronary artery disease, are useful for assessing cardiovascular events [25].

The overall rate of UA amounts to 8.9% in the APACE registry, 11.1% in the RAPID-CPU registry, and 17% in the PLATO trial [26,27,28]. However, in a prospective survey of over 10,000 patients, the proportion of UA was 41.9% [29]. Interestingly, the more sensitive the cTn assay used, the lower the prevalence of UA. In the RAPID-CPU registry, CA and revascularization were performed at a rate of 71.8%. In other registries, the revascularization rate in UA varies and ranges from 21% to 78.3% [14,30].

The rates of CA and revascularization (PCI, CABG) were very high in our study. All 3668 (100%) patients received invasive CA. The revascularization rate was high at 71.3%, probably given that it was in a group of high-risk patients (a high percentage of patients with previous PCI procedures, high LDL cholesterol levels, and smokers).

It is noteworthy that the patients who did not undergo revascularization were more likely to have pre-existing CAD and prior coronary revascularization procedures (PCI and CABG; 49.29% vs. 34.38%, *p* < 0.001) [31]. Moreover, a positive history of heart failure or ischemic stroke was more common in the control group.

The above facts may indicate a different approach being required for the non-revascularization group of patients, including their quicker qualification for invasive procedures based on their medical history and the risk of an impending heart attack. The paradox in the fact that patients with greater comorbidities were less likely to undergo revascularization was also observed by other authors [32]. This suggests that physicians place greater weight on comorbidities (previous MI and revascularizations) and the associated risks.

Among the factors that predicted revascularization due to significant coronary artery stenosis in the multifactorial analysis model were: stenocardial pain of class III, male gender, total cholesterol above 155 mg%, LDL above 87 mg%, MCV below 90.9 fl, PLT above 210 × 10^3^/mm^3^, PT less than or equal to 14.2 s, RDW below 11.6%, and TSH below 1.05 IU/mL.

Stenocardial pain of class III, recognized as severe angina, was significantly more common in the revascularization group. The above result highlights how significant clinical examination is in the diagnosis of UA. The more severe the angina, the greater likelihood of ischemia requiring revascularization in the context of significant coronary stenoses.

Similarly, the male gender was an independent predictor of revascularization caused by significant coronary stenosis. Other authors also found an association between the male gender and obstructive coronary artery disease [33,34].

It was also found that RDW and MCV were significantly lower in patients who underwent revascularization. Geng et al. demonstrated that the baseline RDW was closely associated with in-stent restenosis at follow-up in patients with UA pectoris who underwent successful percutaneous coronary interventions with drug-eluting stents [35]. In the study of Gul et al., RDW was associated with long-term cardiovascular mortality in NSTEMI and UA [36]. In another study, elevated RDW was also an independent predictor of hospital readmission in patients with UA (hazard ratio: 1.35 (95% confidence interval (CI): 1.02–1.79), *p* = 0.033) [37].

Current research has also shown that increasing MPV is associated with MI and UA, while a rising platelet volume is related to an increased risk of mortality due to CVDs [38,39,40]. In the study of Sun et al., low MCV predicted a high risk of in-stent restenosis [41]. Decreased MCV is associated with microcytic anaemia, iron deficiency, and inflammation, which contributes to atherosclerosis [42].

The ROC curve analysis indicated that the factors associated with revascularization due to significant coronary stenosis included the parameters linked to hyperlipidemia (total cholesterol levels > 155 mg/dL, HDL-cholesterol level < 54 mg/dL, LDL-cholesterol level > 87 mg), as well as hematological indices such as MCV < 90.9%, MPV < 8.8%, PLT levels > 210 × 10^3^/mL, prothrombin time < 14.2 s, RDW level < 11.2%, and TSH level < 1.05 µU/mL. However, the sensitivity and specificity of these parameters were limited due to the multifactorial characteristics of the atherosclerotic coronary lesions.

With regards to the prevalence of LBBB and RBBB, both exhibited a significantly higher occurrence in the control group. The bundle branch blocks in the control group were more frequently detected, given the greater tendency of primary care physicians to recognize ACS and refer such patients to the hospital even if the symptoms are not very severe. The same applies to a positive history of myocardial infarction, coronary angioplasty, heart failure, or ischemic stroke.

Other research has highlighted the role of other tools that may help select patients for CA and revascularization procedures in patients with UA. For example, new biomarkers, selected using machine learning and metabolomics techniques, may be used to improve the clinical diagnosis of UA [43,44]. Furthermore, an artificial neural network employing simple, easily available clinical variables may be used to non-invasively identify a group of patients with chest pain without obstructive CAD [45]. Our study, nevertheless, has demonstrated that the high percentage of patients with significant coronary artery stenosis requiring revascularization due to UA are well qualified for invasive procedures.

### Limitations of the Study

There are several limitations to this work: The revascularization/no revascularization analyses were retrospective and single-centered, which makes them subject to a potential bias. The troponin measurements displayed variability within the study group. The extent of new cardiac troponin (cTn) increases depended on the analytical sensitivity of both the current (standard) assay in use (2012–2014) and the high-sensitivity assay (2014–2016) scheduled for implementation. Essentially, the transition from CKMB and early cTn assays to innovative high-sensitivity (hs-cTn) assays may have led to a notable increase in the positivity rate (values > 99th percentile), coupled with a decrease in the incidence of unstable angina (UA). However, when shifting from sensitive contemporary cTn to hs-cTn assays, this change may have been mitigated or absent [46].

The diagnosis of UA relies on the subjective decision of the attending physician. There is also a possibility that the interpretations of a patient’s symptoms may differ markedly between clinicians. Perhaps it would be advisable to pay more in-depth and detailed attention to imaging tests of cardiac ischemia, which would precede a referral to CA. Additionally, coronary artery stenoses were assessed by the operating physician, with no independent assessors reviewing the lesions. This may indicate the possibility of interpersonal differences in the lesions’ interpretation.

Unfortunately, the Fractional Flow Reserve (FFR) assessment in all the cases was hindered by the restricted availability of the procedure in Poland in 2012–2016, which coincided with the execution of the project. All the patients followed a prescribed medical therapy according to the guidelines for managing NSTE-ACS. However, detailed data on the administered pharmacotherapy were not available. This limitation is inherent in retrospective studies, and such information is excluded from the analysis.

## 5. Conclusions

The data presented indicate that UA is still a challenging diagnosis and a significant clinical problem in daily practice. In the registry shown, the proportion of patients with UA among all the ACS patients as well as the percentage of revascularization were very high. It was found that severe angina symptoms, male gender, and hyperlipidemia were independent predictors of revascularization in UA. Paradoxically, patients with a greater burden of comorbidities (previous MI, previous revascularization, intraventricular conduction disorders) were less likely to undergo revascularization.

## Figures and Tables

**Table 1:** Baseline characteristics of the study groups with UA.

Parameter	Unstable Angina with Revascularization *n* = 2615	Control Group *n* = 1053	*p*-Value
Age (years)	65.7 ± 9.3	66.6 ± 9.1	*p* = ns
BMI (kg/m^2^)	28.0 ± 4.7	28.5 ± 4.9	*p* = ns
Male gender (%)	70.16	63.13	*p* = ns
Hypertension (%)	93.8	93.64	*p* = ns
DM type 2 (%)	25.43	23.93	*p* = ns
DM type 1 (%)	0.42	0.19	*p* = ns
Stenocardial pain class I (%)	0.54	0.95	*p* = ns
Stenocardial pain class II (%)	80.84	92.21	*p* = ns
Stenocardial pain class III (%)	18.35	6.74	*p* < 0.001
Stenocardial pain class IV (%)	0.27	0.09	*p* = ns
Chronic Kidney Disease (CKD) (%)	3.56	3.51	*p* = ns
Peripheral Artery Disease (PAD) (%)	6.0	7.12	*p* = ns
Previous MI (%)	20.42	27.45	*p* < 0.001
Previous PCI (%)	34.38	49.29	*p <* 0.001
Previous CABG (%)	8.3	17.47	*p <* 0.001
Stroke (%)	3.17	4.75	*p* = 0.022
Current Smoking (%)	18.45	13.64	*p* < 0.001
Family history of CAD	25.74	30.96	*p <* 0.001
ECG findings Sinus rhythm	71.14	72.46	*p* = ns
LBBB	1.53	3.23	*p* = 0.002
RBBB	0.65	1.9	*p* = 0.001
AF	2.6	3.8	*p* = ns
Heart Rate (bpm)	73.0 ± 13.7	73.1 ± 14.4	*p* = ns
SBP (mmHg)	123.8 ± 16.1	123.9 ± 16.0	*p* = ns
DBP (mmHg)	81.1 ± 11.0	80.7 ± 10.6	*p* = ns
ALT (U/L)	29.9 ± 42.5	28.1 ± 23.0	*p* = ns
AST (U/L)	29.1 ± 56.8	26.7 ± 25.1	*p* = ns
Urea (mg/dL)	40.5 ± 16.3	41.8 ± 18.7	*p* = ns
Na^+^ (mmol/L)	140.9 ± 2.9	141.1 ± 2.6	*p* = 0.033
K^+^ (mmol/L)	4.4 ± 0.4	4.4 ± 0.4	*p* = ns
TSH (µU/mL)	1.5 ± 1.5	1.7 ± 1.6	*p* = ns
Cholesterol (mg/dL)	182.1 ± 53.7	172.9 ± 49.3	*p* < 0.001
TG (mg/dL)	146.3 ± 107.5	144.0 ± 104.2	*p* = ns
LDL (mg/dL)	112.7 ± 47.1	103.4 ± 43.6	*p* < 0.001
HDL (mg/dL)	51.1 ± 14.5	52.6 ± 16.0	*p* = 0.019
Creatinine (mg/dL)	1.05 ± 0.5	1.04 ± 0.5	*p* = ns
GFR (mL/min)	76.8 ± 21.1	75.8 ± 20.3	*p* = ns
CRP (μg/mL)	1.8 ± 3.8	1.3 ± 3.1	*p* = ns
Fibrinogen (mg/dL)	410.9 ± 98.9	408.6 ± 104.5	*p* = ns
APTT (s)	31.6 ± 11.8	31.8 ± 9.9	*p* = ns
HbA1c (%)	7.0 ± 1.5	6.7 ± 1.3	*p* = ns
Haemoglobin (g/dL)	14.4 ± 1.6	14.3 ± 1.6	*p* = ns
HCT (%)	42.7 ± 4.5	42.5 ± 4.3	*p* = ns
RDW (%)	12.3 ± 1.2	12.4 ± 1.2	*p* < 0.001
MCV (%)	91.1 ± 4.9	91.6 ± 5.4	*p* = 0.013
PLT (10^3^/mL)	233.3 ± 67.9	233.1 ± 73.1	*p* < 0.001

The data are expressed as mean ± SD or as percentage. Abbreviations: BMI = body mass index; CRP, C-reactive protein; MCV—mean corpuscular volume; HCT—hematocrit; GFR, glomerular filtration rate; PLT—platelets; hs-TnT, high-sensitive troponin T; CABG = coronary artery bypass grafting; DBP = diastolic blood pressure; RBBB—right bundle-branch block; LBBB—left bundle-branch block; MI = myocardial infarction; PCI = percutaneous coronary intervention; SBP = systolic blood pressure; AF—atrial fibrillation; CKD—chronic kidney disease; CAD—coronary artery disease; APTT—activated partial thromboplastin time; ECG—electrocardiography; DM—diabetes mellitus; TC—total cholesterol; TG—triglycerides. Stenocardial pain class I—mild, class II—moderate, class III—severe, class IV—very severe.

**Table 2:** Predictors of revascularization in patients with UA—results from multivariate logistic regression modeling using the backward stepwise logistic regression method.

Variable	OR	95% CI	*p*-Value
LBBB	0.51	0.29–0.89	0.018
RBBB	0.33	0.15–0.71	0.004
Male gender	1.49	1.21–1.84	<0.001
PLT > 210 (10^3^/mL)	1.34	1.10–1.64	0.003
LDL > 87 mg/dL	1.49	1.22–1.82	<0.001
PT ≤ 14.2 s	1.35	1.04–1.75	0.023
MCV < 90.9%	1.27	1.05–1.55	0.015
Stenocardial pain class III	2.78	2.01–3.86	<0.001
RDW < 11.6%	1.31	1.03–1.66	0.025
Previous CABG	0.66	0.49–0.89	0.008
TSH < 1.05 uIU/mL	1.31	1.07–1.61	0.009
Family history CAD	0.49	0.39–0.61	<0.001

The data are shown as odds ratio (OR) with 95% confidence intervals. Abbreviations: LBBB = left bundle-branch block; RBBB = right bundle-branch block; PCI = percutaneous coronary intervention; PLT—platelets; CCS—Canadian Cardiovascular Society; LDL—low density lipoprotein; PT—prothrombin time; RDW—red cell distribution width; TSH—thyroid stimulating hormone; MCV—mean corpuscular volume; CABG—coronary artery bypass grafting; CAD—coronary artery disease.

## Data Availability

The data presented in this study can be made available upon request from the corresponding author.
